# Preserving Genome Integrity during the Early Embryonic DNA Replication Cycles

**DOI:** 10.3390/genes10050398

**Published:** 2019-05-24

**Authors:** Chames Kermi, Antoine Aze, Domenico Maiorano

**Affiliations:** 1 Laboratoire Surveillance et Stabilité du Génome, Institut de Génétique Humaine, UMR9002, CNRS, Université de Montpellier, 34090 Montpellier, France; kermi@stanford.edu (C.K.); antoine.aze@igh.cnrs.fr (A.A.); 2 Department of Chemical and Systems Biology, Stanford University School of Medicine, 318 Campus Drive, Stanford, CA 94305-5441, USA

**Keywords:** DNA damage, DNA damage tolerance, replication stress, *Xenopus laevis*, *Drosophila melanogaster*, zebrafish, *Caenorabditis elegans*, mouse embryonic stem cells, iPSCs

## Abstract

During the very early stages of embryonic development chromosome replication occurs under rather challenging conditions, including a very short cell cycle, absence of transcription, a relaxed DNA damage response and, in certain animal species, a highly contracted S-phase. This raises the puzzling question of how the genome can be faithfully replicated in such a peculiar metabolic context. Recent studies have provided new insights into this issue, and unveiled that embryos are prone to accumulate genetic and genomic alterations, most likely due to restricted cellular functions, in particular reduced DNA synthesis quality control. These findings may explain the low rate of successful development in mammals and the occurrence of diseases, such as abnormal developmental features and cancer. In this review, we will discuss recent findings in this field and put forward perspectives to further study this fascinating question.

## 1. Introduction

Preserving genome integrity during the cleavage stage of early embryogenesis is primordial to the embryo given its potential to generate multiple, distinct cell lineages. Mutations that occur during the very early embryonic replication cycles may be fixed and inherited in differentiated cells. This may constitute a potential risk to develop genetic diseases, amongst which cancer, through oncogene activation and/or tumor suppressor silencing [1]. Strikingly, a number of observations indicate that embryonic cells display several signs of genomic instability, to a level comparable to that observed in cancer cells. The molecular bases of such genetic instability are currently unclear, as it is also unclear how the embryos can cope with this potential source of DNA damage, yet leading to the generation of viable and healthy organisms. Nonetheless, the rate of successful development is in general rather low (between 25% and 30% for humans [2] and between 21% to 50% for some frog species in the wild [3], and references therein). Animals that reproduce themselves by external fertilization, such as anamniotes, compensate for this drawback by generating a large number of embryos, thus increasing the chances to end up with few healthy individuals that survive to predators and adverse environmental conditions. The development of these embryos is usually fast. The situation is different in amniotes, such as mammals, where fertilization is internal, development slower, and a restrained number of embryos are produced.

To which extent a low rate of healthy development is a consequence of a faulty control of genome integrity during early embryogenesis remains an open question. The very first DNA replication cycles occur in a particular molecular environment, that is, the fertilized egg, which is very different from that of a somatic cell, and can greatly influence the response to genomic insults. We aim to compile the most recent works that emphasize these critical processes mostly in vertebrates, but we will also refer to some general concepts learned from metazoans invertebrates that we believe contribute to a better comprehension of vertebrates’ mechanisms governing genome integrity.

## 2. Embryonic DNA Replication

**Figure 1:**
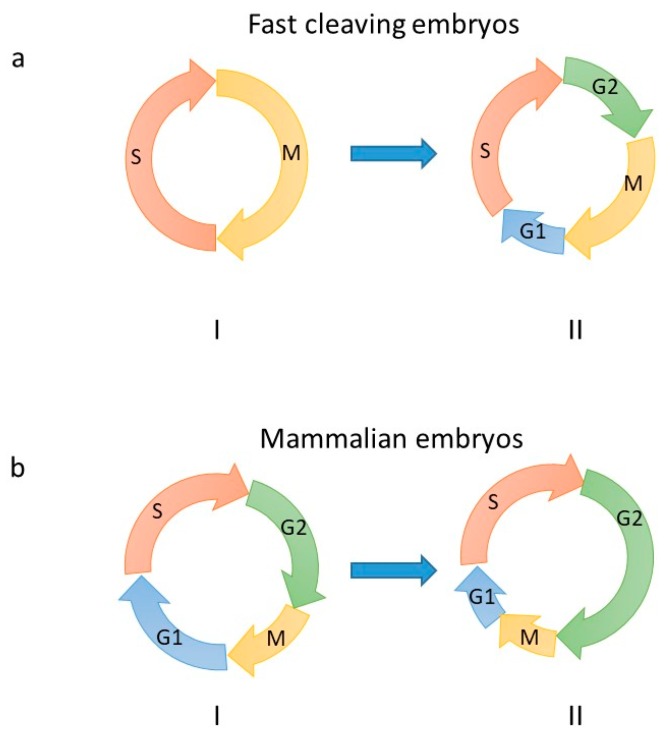
Embryonic cell cycles. Cell cycle remodeling during the early stages of embryonic development. (**a**) Pre-MBT (I) and post-MBT (II) cell cycle of fast cleaving embryos (*Caenorabdtis elegans*, zebrafish, *Drosophila* and *Xenopus*). Onset of G1- and G2-phases appear at different developmental times depending on the organism. In zebrafish, G1 and G2 occur at MBT. In *Xenopus* and *Drosophila* G2 appears at MBT, while the G1-phase is observed upon gastrulation. (**b**) First (I) and second (II, Zygotic Genome Activation (ZGA) occurs at this stage in mouse) cell cycle of pre-implantation mammalian embryos. The cell cycle length of mice gastrula cells is much more contracted, notably S-phase only lasts 2 h compared to an average of 6 h in pre-implantation embryos (reviewed in [11]). Of note, length of cell cycle phases may be different between mouse and human pre-implantation embryos. Arrows indicate the direction of cell cycle progression. Letters indicated cell cycle phases.

During the first replication cycle, the egg and sperm chromosome replicate separately, then they fuse at the end of DNA synthesis to generate the embryo’s nucleus. DNA synthesis in the fertilized egg occurs within a special environment. The volume of an egg is between five and 50 times larger than that of a somatic cell, depending on the species. Such a feature affects the nucleus-to-cytoplasm (N/C) volume ratio, which is low in the early embryo. In addition, most of the cellular proteins in the egg are present in large excess, thus counteracting the natural repression of transcription, probably induced by an excess of histones pool that compete out transcription factors for binding to their chromatin targets [4,5,6]. A consequence of this molecular microenvironment is a strong contraction of the cell cycle. In fast cleaving embryos, such as *Caenorabidtis elegans*, *Drosophila melanogaster*, *Danio rerio* (zebrafish) and *Xenopus laevis*, this accounts for short, or in some cases inexistent, gap phases (G1- and G2-phases), and a highly contracted S-phase (between four and 20 min Figure 1). During the cleavage stages of *Xenopus* development, DNA replication occurs within micronuclei-like structures (the karyomeres) already at the end of M-phase before nuclear reconstitution. Karyomers then disappear at the time of zygotic genome activation (ZGA), the mid-blastula transition (MBT, [7]). In the *Xenopus* and *Drosophila* early embryos, initiation of DNA replication does not show any specificity and occurs randomly throughout the genome, though respecting a constant spacing between replication origins [8,9]. Fork speed is increased three-fold compared to somatic cells, clusters of replication origins are abundant and fire synchronously [9]. This regulation ensures complete genome replication within a very short S-phase. At MBT, activation of the S-phase checkpoint, transcription activity and/or chromatin remodeling imposes a temporal order of replication origin firing and dictates DNA replication origin specificity [9,10] (see below). The very early mammalian embryonic cell cycle also displays contracted G1- and G2-phases, however the duration of S-phase is more similar to that observed in somatic cells (between 4 and 8 h).

**Figure 2:**
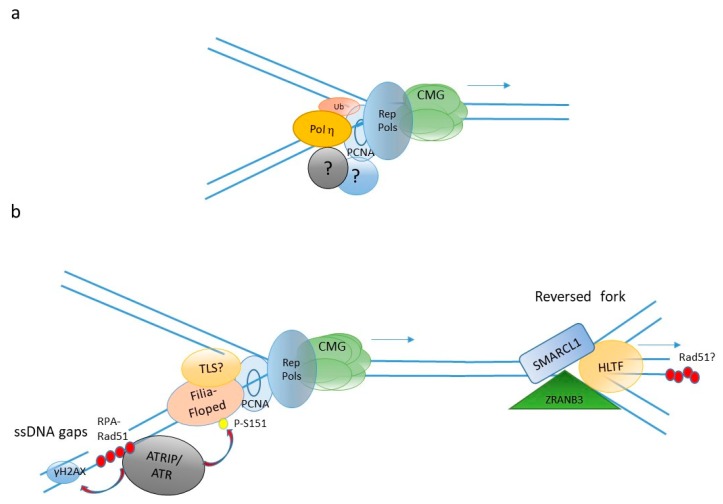
Embryonic DNA replication fork features. Schematic representation of DNA replication forks in (**a**) fast cleaving embryos and (**b**) mouse embryonic stem cells. Question marks indicate so far unidentified proteins including Y-family translesion synthesis (TLS) pols. Rep Pols indicates replicative DNA pols. Ub indicates ubiquitin. CMG indicates the replicative helicase. Arrows indicate the direction of translocation of the fork.

The anatomy of the DNA synthesis machinery of early embryos is very similar to that of somatic cells (reviewed in this special issue [12]), yet with few, but important differences (Figure 2). In *Xenopus* embryos there is evidence for a DNA damage-tolerant replisome that contains at least one specialized, translesion synthesis (TLS) DNA polymerase, polη, in addition to the three canonical replicative DNA polymerases, α, δ and ε [13]. This specific architecture is generated by monoubiquitination of the DNA polymerase δ-associated factor PCNA catalyzed by the Rad18 (E3)-Rad6 (E2) ubiquitin ligase complex (reviewed in this special issue [14]) which is abundant in early *Xenopus* embryos [13]. The presence of TLS polη into the replisome may help to minimize replication fork stalling in front of DNA lesions and/or chromatin structures that are difficult to replicate. This strategy, coupled to a high density of replication origins, ensure complete genome replication within a very short S-phase (20 min in *Xenopus*). Because TLS polymerases are error-prone [15], it implies that in the early embryo fast DNA synthesis may be mutagenic, although this point has not yet been experimentally proven. Maternal isoforms of at least two DNA replication proteins, the replication initiation factor CDC6 [16] and the MCM6 subunit of the MCM2-7 replicative helicase complex [17], have been reported in *Xenopus*. Maternal MCM6 (mMCM6) lacks a carboxyl-terminal extension and a cyclin dependent kinases (CDK) phosphorylation consensus site compared to the zygotic MCM6.

Two isoforms of CDC6, A and B, are present in the egg, encoded by two distinct genes, but only the A isoform appears to participate to DNA replication in very early embryogenesis. The expression of CDC6A declines close to the MBT, while CDC6B is also found at later developmental stages replacing CDC6A. At the protein level, the two CDC6 isoforms differ in their amino-terminal part for the presence of both regulatory signals for phosphorylation by S-phase CDKs and a destruction box that targets CDC6 for degradation. The zygotic CDC6B isoform contains a “KEN box” sequence required for cell cycle-regulated proteolytic destruction. However, this sequence is divergent in the maternal CDC6A isoform, which is refractory to proteolysis and remains stable throughout the early embryonic cell cycles. Further, a second activator of the CDC7 S-phase promoting kinase, the DRF1 protein (for DBF4-related factor 1), has been described in *Xenopus*. DRF1 and not DBF4, is required for DNA synthesis in early embryogenesis. Close to MBT, DRF1 levels drop sharply [18,19]. Recent evidence shows that destabilization of DRF1 at MBT depends upon activation of the checkpoint kinase CHK1 ([20] and see Section 3). DBF4, whose level does not change throughout development, replaces DRF1 in the post-MBT embryo. It is currently unclear why DBF4 is excluded from the replication machinery from fertilization until MBT. Recently, a requirement for early cleavages DNA replication of a large chromatin remodeling complex xNuRD, has been highlighted in early *Xenopus* embryos. Before MBT, this complex seems to ensure the function of small non coding Y-RNAs in replication initiation observed after MBT [21,22], however the molecular mechanism involved remains currently unclear [23]. Furthermore, while Y-RNAs function appears to be conserved in somatic human cells, NuRD activity is not shared.

A screen by iPOND (immunoPrecipitation Of Nascent DNA) has identified two proteins, Filia and Floped, as specific components of the replication fork in mouse embryonic stem (ES) cells (Figure 2b), while these proteins are undetectable in differentiated cells [24]. The authors showed that both proteins are important to facilitate replication fork restart stalled by the nucleotides synthesis inhibitor hydroxyurea. Their function in replication fork restart requires ATR phosphorylation, which facilitates recruitment of the BLM helicase and boosts activation of the S-phase checkpoint. Depletion of Fila and Floped results in genomic instability and resistance to apoptosis, leading to malignant transformation. Recently, analysis of DNA replication by electron microscopy has revealed that mouse ES cells contain a high abundance of single-strand (ss) DNA gaps and reversed forks [25]. These latter are replication intermediates formed when replication forks are remodeled into a four-way junctions structure by the action of specific DNA translocases including HLTF, SMARCL1, and ZRANB3 [14,26] (for review, and Figure 2). Consistent with this observation, spontaneous foci of RPA and RAD51 ssDNA binding proteins, as well as foci of γH2AX, a non-specific marker of DNA damage and replication stress (see below), can be observed in the nuclei of ES cells [25,27]. Altogether, these observations suggest that DNA replication is mouse ES cells may be incomplete and that fork reversal may be a mechanism to cope with DNA damage-induced replication stress.

## 3. Preserving Genome Integrity during Early Embryogenesis Replication Cycles

Coordination of cell cycle phases is a key aspect in response to DNA damage. Somatic cells possess feedback mechanisms, known as cell cycle checkpoints, whose function is to slow down, or arrest the cell cycle when the integrity of the DNA is compromised [28]. This regulation restrains cell division to avoid accumulation of mutations or genomic alterations in the daughter cells, thus ensuring faithful transmission of the genetic information. DNA integrity is sensed by three major apical protein kinases belonging to the PI3-like family, namely DNA-PKcs, ATM and ATR [29]. Their activation by DNA damage, or even by changes in chromatin structure (for DNA-PKcs and ATM), generates a phosphorylation cascade leading to phosphorylation of hundreds of downstream substrates, including the CHK1 and CHK2 protein kinases, the p53 tumor suppressor and the H2AX histone variant (γH2AX being its phosphorylated form). This signaling can even span beyond the nucleus and influence the general metabolism of the cell [30]. The end point of these signaling pathways are the master cell cycle regulators, such as members of the CDC25 protein family (A, B and C), CDKs, as well as the major S-phase promoting kinase, DDK (for DBF4-dependent kinase). Activation of these kinases also stimulates DNA repair and DNA damage tolerance pathways. ATR is currently considered as the principal sensor of replication fork progression [31], but recent evidence also implicates DNA–PKcs in signaling DNA damage at stalled forks synergistically or redundantly with ATR [32,33,34]. Stalling of replication forks can lead to the formation of excess ssDNA, which constitutes a major signal for ATR activation. ATR activation depends upon accumulation of replication intermediates on the lagging strand [35,36]. These structures are made of a 5′ ssDNA–dsDNA junction recognized by the RF-C^Rad17^ complex that, in turn, loads onto this structure the checkpoint clamp complex made of RAD9-HUS1-RAD1 proteins (9-1-1 complex) [37,38]. Loading of 9-1-1 is essential to tether interaction between the TOPBP1 protein, an ATR activator, with the ATR-ATRIP complex leading to ATR activation ([39] for review). A second ATR activator, the ETAA1 protein, has recently been identified [40,41,42,43]. ETAA1 interacts with the major ssDNA binding protein complex RPA and functions in activating ATR independently of TOPBP1. Mutations of the ETAA1 gene are associated with susceptibility to pancreatic cancer [44]. There are situations in which replication fork stalling does not generate excess ssDNA and therefore ATR is not immediately activated. These include hard to replicate DNA regions (prone to form alternative DNA structures for instance), but equally DNA lesions that inhibit progression of the replicative helicase (reviewed in [45]). Prolonged replication fork arrest in these conditions can generate DNA double strand breaks (DSBs) that if resected, generate ssDNA and ultimately induce ATR activation.

Several reports have shown that early embryos, as well as embryonic stem cells, lack a stringent control of cell cycle checkpoints (Table 1), in that DNA synthesis and/or cell division continues in the presence of damaged DNA or faulty chromosomes alignment on the mitotic spindle [46,47,48,49,50,51]. In *Drosophila*, maternally supplied ATR (Mei-41) is essential during syncytial nuclear divisions, and null mutant flies in this gene never reach the cellular stage [52,53]. *Drosophila* CHK1 (Grapes) [54] is essential to normally progress through MBT, as well as in *C. elegans* [55], by reducing the activity of the mitotic activator CDK1, and therefore preventing mitotic catastrophes as a consequence of a lengthened S-phase [56]. DNA damage generated by Grapes mutants is sensed by CHK2 (Mnk) leading to G2 arrest [57]. Interestingly, the *Drosophila* CDK1 inhibitor WEE1 kinase (Dwee1) is also required to complete the early embryonic cell cycles and genetically interacts with Mei-41 and Grapes, showing that it functions in the same regulatory pathway [58]. Altogether, these observations show that the ATR-CHK1-CHK2 pathway is essential to preserve genome integrity in the developing embryo. A relaxed spindle assembly checkpoint has been reported in *C. elegans* [59], zebrafish [60] and *Xenopus* [50,61] which is affected by cell size, age but also cell fate. Further, in the early *Xenopus* [62,63], human and bovine embryo [64,65], pathways leading to apoptosis activation appear to be suppressed.

**Figure 3:**
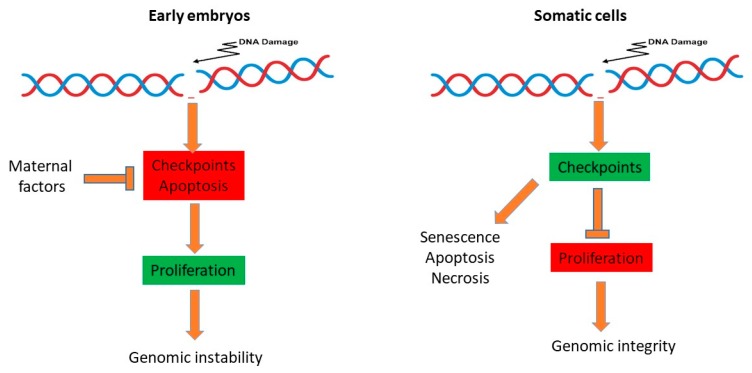
Differences in the regulation of proliferation in embryos versus somatic cells. Inefficient checkpoint activation, suppression of apoptosis and a high contracted cell cycle promotes rapid and unchecked proliferation in the early embryo leading to the generation of a certain degree of genomic instability. In somatic cells, DNA damage is readily monitored by checkpoints that slow down cell proliferation to facilitate DNA repair, and as such preserve genome integrity, leading, when necessary, to permanent cell cycle arrest (senescence) or cell death (apoptosis, necrosis).

What is so special about embryonic cells that make them so different from somatic cells? Inefficient checkpoint activation and suppression of apoptosis in the early embryo is intrinsically linked to a highly contracted cell cycle, which is probably imposed by the high abundance of almost all cellular proteins accumulated in the egg during the maturation process. This adaptation has probably evolved to guarantee execution of the first embryonic cleavages, even under unfavorable environmental conditions. At the embryonic stem cells stage, a contracted G1 phase appears to be a necessity to maintain the pluripotency state, at least in mice [66,67]. This context translates into some inevitable constraints on the integrity of the genome. Fast cleaving embryos may benefit from having a contracted and unchecked cell cycle, because a rapid development is probably important for survival in the external environment. For example, there is evidence that in frogs, predators’ attack can accelerate the speed of hatching [3]. During the cleavage stage, blastomers carrying DNA damage rely on maternal factors to survive within the embryo, at least until later stages of development when a more strict control of genome integrity occurs, namely at the MBT and the pre-implantation stage in mammals (Figure 3, [68,69]). However, it is not clear how much DNA damage is inherited in the post MBT or pre-implantation embryos, and what the consequences of this leftover damage can be in the adult organism. Altogether, these observations suggest that compared to somatic cells, DNA damage is tolerated in the early embryo, at the expenses of reduced genomic integrity in order to accelerate the early steps of development.

### 3.1. G1/S Checkpoint

This checkpoint operates at the G1- to S-phase transition of the cell cycle and monitors the integrity of the DNA before cells enter S-phase. Traditionally, the tumor suppressor protein p53 has been considered as the major downstream effector of this checkpoint as the target of the ATM and CHK2 protein kinases. P53 has several distinct functions, but its main activity relies on the transcriptional activation of hundreds of downstream genes including the CDK inhibitor p21, several DNA repair and DNA damage tolerance genes and regulators of apoptosis ([70], for review). Fast cleaving embryos and mouse ES cells lack an efficient G1/S checkpoint, so that they cannot arrest in G1 in the presence of DNA damage. In *Xenopus*, p53 was previously shown to translocate into the nucleus of the early embryo [71], while mouse ES were initially reported to be deficient in p53 activity [72,73]. Later studies have demonstrated that in these latter p53 is phosphorylated, is capable of translocating into the nucleus, binding to chromatin and transactivating downstream target genes following DNA damage [67,74,75,76,77]. These findings demonstrate that inefficient p53 function is not the cause of a relaxed G1/S checkpoint. More recent findings have revealed that a high level of the CDC25A protein phosphatase, a major CDKs regulator and a CHK1 downstream effector, modulates the efficiency of the G1/S checkpoint in mouse ES cells. High levels of CDC25A in these cells are maintained by the DUB3/USP17L2 ubiquitin hydrolase through the removal of polyubiquitin chains. The DUB3 gene is itself a target of two pluripotency factors, ESRRβ and SOX2, and its expression is developmentally regulated, being very rapidly downregulated upon the onset of differentiation [67]. Hence, restraining the G1/S checkpoint appears to be an intrinsic feature of mouse ES cells, untimely linked to the pluripotency state. This conclusion is supported by the observation that maintenance of pluripotency is tightly linked to cell cycle length [66]. A different situation was reported in human ES cells, in which the G1/S checkpoint appears to be functional, while the S-phase checkpoint seems to be compromised [78,79]. This difference may be due to differences in the cell cycle of mouse and human embryos and differences in the wiring of pluripotency. For instance, while in mouse embryos ZGA occurs at the two-cell stage, in human embryos ZGA occurs at the eight-cell stage (Table 1). Consistent with this hypothesis, it has been recently reported that primates appear to have less robust genome surveillance mechanisms than rodents [80].

### 3.2. S-Phase Checkpoint

This checkpoint is activated following replication forks slow down or arrest and depends mostly on the ATR kinase. Fast cleaving embryos clearly lack this checkpoint. Original observations in *Drosophila* [81], *C. elegans* [82], zebrafish [83] and *Xenopus* [84,85,86,87] have shown that early embryos continue to divide in the presence of DNA synthesis inhibitors. For instance, microinjection of aphidicolin, a competitive inhibitor of replicative DNA polymerases, into pre-syncytial *Drosophila* embryos, inhibited DNA synthesis and slowed down, but did not arrest, embryonic cleavages, while the centrosome cycle was unaffected [81]. Further, soaking fertilized *Xenopus* eggs in aphidicolin, did not slow down embryonic cleavages but embryos were devoid of nuclei, demonstrating efficient aphidicolin uptake into the embryos [87]. A similar result was obtained by direct microinjection of aphidicolin into *Xenopus* two-cell stage embryos [86]. Recently, a more detailed analysis of embryonic cell cycle progression under very similar experimental conditions, showed that aphidicolin can induce a cell cycle delay in pre-MBT *Xenopus* embryos [20], similar to what observed in *Drosophila* [81], although development was not inhibited. Several reports have also shown that in *Xenopus* the developmentally-regulated activation of the S-phase checkpoint can be faithfully reproduced in vitro by varying the amount of nuclei in egg extracts from pre-MBT to MBT levels [13,84,88,89]. Recent evidence has implicated TLS in silencing the DNA damage checkpoint in *C. elegans* [82,90]. In this organism, mutation of the polη gene induced a cell cycle delay upon exposure to DNA damaging agents. More recent evidence in *Xenopus* has confirmed this observation and proposed a molecular mechanism explaining the inefficiency of the DNA damage checkpoint in early embryos. In cleavage-stage *Xenopus* embryos, constitutive recruitment of TLS polη onto replication forks, driven by the Rad18 ubiquitin ligase, minimizes replication fork stalling in front of UV lesions and possibly other DNA insults, thereby limiting the production of ssDNA, which is essential for ATR activation. This configuration is lost close to MBT following a developmentally regulated decline of Rad18 abundance [13]. An inefficient S-phase checkpoint has also been documented in human ES cells, in which exposure to thymidine or cisplatin fails to induce full activation of the CHK1 kinase [78]. The molecular grounds of this regulation are currently unknown. Finally, a developmental-specific control of DNA rereplication has also been reported in mouse ES cells. It was shown that Geminin, an inhibitor of the replication initiation factor Cdt1 [91,92], is essential for viability and inhibition of apoptosis in pluripotent and not differentiated cells [93].

## 4. DNA Repair and DNA Damage Tolerance in the Embryo

DNA carries the genetic information that shapes the life of an organism, however its structure is labile and has a limited chemical stability [94]. Cells have developed sophisticated DNA repair mechanisms to safeguard genome integrity including the four major DNA repair pathways. These include the base excision repair (BER) and the nucleotide excision repair (NER) that remove one or a few damaged DNA bases, the mismatch repair (MMR) that displaces a patch of nucleotides or misaligned DNA, and DSBs repair by homologous recombination (HR) or non-homologous end joining NHEJ. During the process of DNA repair, stress sensors interact with components of the cell cycle machinery to delay or arrest the cell cycle and thus leave enough time to repair. Further, if the level of DNA damage is too high to be repaired, DNA damage sensors could trigger an apoptotic cell death response to eliminate non-viable cells [95]. Although the roles of the DNA repair machinery in the response to genotoxic stress have been studied extensively in cancer models, less is known about their regulation or activity during early embryonic development. Many gaps exist in our current knowledge concerning the precise role and expression timing of several DNA repair genes in the early stages of embryonic development. The observed developmental stage-specific variations in DNA repair gene expression transcripts and proteins point out the complexity of the regulation of these pathways during development.

During very early embryogenesis DNA transcription is inactive, thus the embryo’s ability to repair DNA is dependent on the DNA repair proteins derived from the oocyte [96]. However, there is evidence that the capacity of DNA repair in the oocyte is limited [97,98]. Hence, DNA damage appears to be tolerated until later stages of development, without disturbing the first developmental stages that mainly rely on the presence of maternal RNA. In zebrafish, a specific p53 isoform has been very recently proposed to regulate DNA damage tolerance in embryos when fertilized with damaged sperm. Embryos continue to divide and develop even in the presence of a high level of DNA damage while DNA repair was induced at the MBT [98]. It has been suggested that p53 modulates the onset of apoptosis in the early embryos by repressing the pro-apoptotic factor NOXA and upregulating the anti-apoptotic factor BCL2. Equally, in *Xenopus* embryos apoptosis onset is observed at the MBT upon exposure to ionizing radiation [62,63]. Mouse ES cells spontaneously accumulate ssDNA gaps, as observed by electron microscopy but it is currently unknown whether (and how) these gaps are repaired. These cells also show constitutive high level of γH2AX, RPA and RAD51 nuclear foci indicating the presence of DNA damage [25,27], a phenotype also observed in induced pluripotent stem cells (iPSCs) [99]. Further, mouse embryos lacking the DNA mismatch repair PMS2 protein develop unrepaired replication errors during early cell divisions [100]. Furthermore, mutations that accumulate in MLH1 mismatch repair-deficient embryos do not appear to compromise development, suggesting that early embryos can cope with a high dose of mutations [101]. At later stages, the embryo progressively acquires greater ability to respond to DNA damage by regulating and activating DNA damage control genes [102].

Human embryos display a high incidence of aneuploidy and polyploidy resulting in post-zygotic chromosomal mosaicism (see below). These chromosomal abnormalities might be accounted for deficiencies in recombination repair and are consistent with the observation that eggs have a low DNA repair capacity [103]. However, this possibility is in contradiction with the observation that the pivotal HR repair gene RAD51 is essential for embryo’s viability [104,105]. A possible explanation of this apparent paradox has been provided by observations in mice, describing an important role of RAD51 in protecting replication forks from degradation by the MRE11 nuclease [106]. Consistent with these findings, removal of RAD51 from *Xenopus* egg extracts results in the accumulation of ssDNA gaps [107]. Interestingly, knock out of p53 partially rescued the embryonic lethality of RAD51 null mouse embryos, suggesting that the early lethality phenotype is dependent upon a p53-dependent DNA damage response. Ablating this response allows proceeding further in development, even with a high level of DNA damage. Hence, early embryos may strongly rely on RAD51 function in order to preserve replication forks integrity when challenged by DNA lesions and other forks impediments. Overall, these observations suggest that unlike somatic cells, embryos activate DNA damage tolerance pathways, including translesion DNA synthesis and replication fork reversal, so to cope with high replication stress and genomic insults, and may tolerate DNA damage, by suppressing apoptosis, probably because there is no enough time for repair.

## 5. Consequences of Reduced Genome Surveillance in the Early Embryo

Adaptation to an embryonic environment may increase the risk of propagating mutations into the developing embryo [108]. From previous and current literature, it is clear that early embryos accumulate mutations and genomic imbalances due to inefficient activation of checkpoints that safeguard genome integrity, the most common alteration being aneuploidy [109,110,111]. A high abundance of proto-oncogenes (c-Myc, Cyclin E, CDT1 amongst others), and small molecules in the egg (ribonucleotides for instance) constitute a potential threat to genome integrity. An excess of ribonucleotides may lead to a high level of their incorporation into the DNA thereby inducing replication stress, and there might not be enough time to remove them in the highly contracted S-phase of fast cleaving embryos, or during the short cell cycle of mammalian embryos. Consistent with this possibility, knock-out of RNAseH2, an enzyme that removes ribonucleotides, is embryonic lethal in mice [112]. Recent data now show that mice embryos can actually tolerate a threshold level of ribonucleotides incorporation into the DNA, although this triggers an innate immune response leading to prenatal lethality [113]. A very recent report proposed that replication fork instability in the early mice embryo can cause lethality through the activation of inflammation [114]. This effect appears to be female-specific, since males are protected by the beneficial effect of testosterone that can stimulate replication initiation (licensing), thus rescuing genome instability [115]. This phenomenon is consistent with a previous finding showing that the processing of unstable replication forks generates fragments of ssDNA that move to the cytoplasm and activate the cGAS-Sting cytosolic DNA-sensing pathway which in turn activates the immune response [116]. The origin of constitutive DNA damage of mouse ES cells and iPSCs, and how cells cope with it, is currently unknown. Nevertheless, such a high load of DNA damage and genomic instability strongly impacts the use of these cells in regenerative medicine, since they are prone to form tumors once injected into an organism [117]. A recent report suggests that maintenance of a stable level of several DNA repair proteins, such as ATR and BRCA1, is important to preserve genome integrity in the mouse embryos. It also appears that early embryos require HR repair to preserve replication fork integrity in the absence of external damage [118]. Hence, lowering the expression of these proteins, as obtained in mice by knockout of the transcription-associated kinase CDK12, leads to a spontaneous accumulation of DNA damage and increased apoptosis [119]. The preference for HR repair may be an adaptation to the contracted G1-phase.

**Figure 4:**
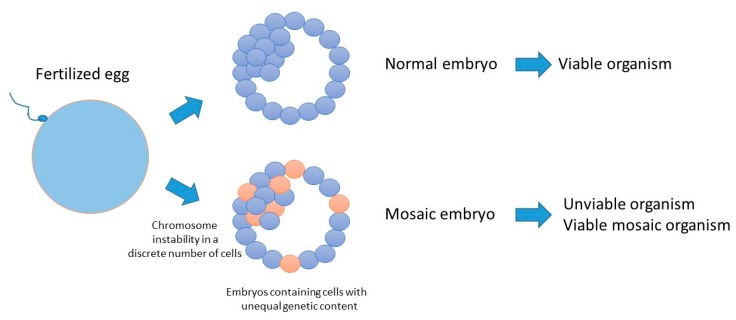
Consequences of chromosome abnormalities in early mammalian embryos. Each circle represent a blastomere, normal (blue) or having a different genetic content (orange).

Errors occurring during the early cleavage stages can generate mosaicism, which is the occurrence of cell populations with non-equal genetic content within the blastocyst at a frequency of about 17%, including polyploidy and aneuploidy [109,120] (Figure 4). Mosaicism can lead to pregnancy failure in humans, due to imbalanced genetic material in the developing embryo, but it may also have no detectable phenotype and lead to the generation of healthy organisms at birth. This latter observation may be explained if cells containing unbalanced genetic context may have been depleted from the inner cell mass of the embryo [121], or if the unbalanced genetic information is rescued and becomes compatible with life. One example of such a rare situation in human is the Dyskeratosis congenital disorder, in which the mosaicism generated by a mutant allele is spontaneously reverted by mitotic recombination. Patients affected by this disorder display skin spots that increase with life, as well as other abnormalities [122]. A number of abnormalities of embryonic origin have been also described in other model organisms. In zebrafish, damaged spermatozoa generate multimalformed embryos, as a consequence of defective chondrogenesis, skeleton morphogenesis, pigmentation, heart morphogenesis, angiogenesis and lymphatic vessels formation [98]. A recent report has highlighted an essential requirement for the humpty dumpty gene in DNA replication and genome integrity of pre-syncytial *Drosophila* embryos [123]. Mutations in its human orthologue *Donson* causes microcephalic dwarfisms [124,125]. Finally, a recent report has revealed the presence of a genome instability process, involving chromosome number variation (CNV), restricted to early human development that may be the consequence of faulty replication fidelity coupled with low processivity [126].

## 6. Conclusions and Perspectives

DNA replication in early embryos occurs under challenging conditions dictated by a special environment constituted by the maternal components of the egg. Early embryos are characterized by rapid cell cycles, restrained control of genome integrity and presence of genomic alterations. In the fast cleaving embryos of *C. elegans* [82,90], *Xenopus* [13] and *Drosophila* (our unpublished observations) there is evidence for the presence of a DNA damage tolerant, error-prone replisome. Because of a highly contracted cell cycle and a very short S-phase, it is possible that if mutations are introduced, there will be very little or no time to detect and correct them by the MMR system. This may occur at later stages of development, namely at MBT, when the cell cycle slows down and the first activation of the zygotic genome is observed. However, at this stage several mutations may have already been fixed during the embryonic replication cycles. If this is the case, this may constitute a novel and unexpected source of genetic diversity occurring in the early embryo that could contribute to the polymorphisms of the adult organism. Introduction of mutations can have adverse effects. It can be beneficial by increasing diversity, as observed during the meiotic process in gametes, but it can also be deleterious by fueling genomic instability. Notwithstanding, the consequences of relaxed genome integrity controls in the early embryos remain to be fully addressed.

Interestingly, embryonic features are also strikingly common to cancer cells (Table 2), suggesting an interesting parallel between these two cellular states. Can cancer ultimately be considered as an awakening of an early embryonic state in differentiated tissues? Several lines of evidence point to such a possibility. Relaxed DNA damage checkpoints due to mutations in genes controlling this signaling pathway are a feature of cancer [127].

**Figure 5:**
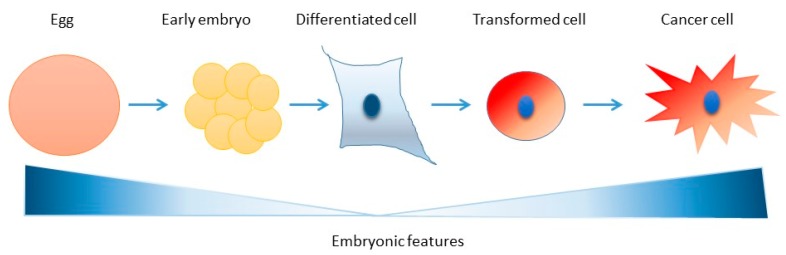
Awakening of embryonic features in cancer cells? Cartoon showing the degree of appearance of embryonic features in cells during the process of malignant transformation.

Aggressive, poorly differentiated cancer cells are often characterized by an embryonic gene expression signature [128]. The epithelial to mesenchymal transition (EMT), currently implicated in metastasis and therapeutic resistance of cancer, recapitulates morphological changes and the migration phenotypes observed in cells during early development. Interestingly, the expression of embryonic markers is linked to EMT [129]. Cancer cells are also characterized by a loss of contact inhibition, the ability to reprogram into an undifferentiated state and for showing resistance to DNA damage (Table 2 and Figure 5). Finally, cancer cells present genomic instability and a mutator phenotype [130]. Hence, studying the regulation of the network that preserve genome integrity during the replication cycles of early embryos may lead to a better understanding of cancer biology.

## Figures and Tables

**Table 1:** Genome integrity checkpoint and zygotic genome activation during early embryogenesis. The table summarizes the presence (+) or absence (−) of the indicated genome integrity checkpoints observed in fast cleaving early embryos (*C. elegans*, *Drosophila*, zebrafish, and *Xenopus*), as well as in mouse and human embryos. n.d.: not determined; SAC: spindle assembly checkpoint. ZGA refers to the early waves of transcription occurring at MBT in *Drosophila*, zebrafish and *Xenopus laevis*.

Checkpoint	Fast Cleaving Embryos	Mouse	Primates	Human
G1/S	−	−	n.d.	+
S	−	+	n.d.	+
G2/M	+	+	+	+
SAC	+/−	+	n.d.	+
DNA damage checkpoint activation	32-cells (*Xenopus laevis*, [13])	2-cells	4–8 cells	4–8 cells
ZGA	3000–6000 cells	2-cells	4–8 cells	4–8 cells

**Table 2:** Features shared between early embryos, normal non-cancer and cancer cells. Unstructured chromatin features can be variable in cancer cells, with certain cancer cells having more structured chromatin than others do. N/C: nucleus to cytoplasm volume ratio. This can be variable in different cancer cells types and at different stages of malignant transformation.

Features	Embryos	Normal Cells	Cancer Cells
High proliferation rate	YES	NO	YES
Contact inhibition	NO	YES	NO
Migration	YES	NO	YES
Undifferentiated state	YES	NO	YES
Unstructured chromatin	YES	NO	YES/NO
Genetic instability (CNV)	YES	NO	YES
Inhibition of apoptosis	YES	NO	YES
Resistance to DNA damage	YES	NO	YES
Mutator phenotype	?	NO	YES
N/C ratio	Low	High	Low/High
